# Effects of heart rate variability biofeedback training in athletes exposed to stress of university examinations

**DOI:** 10.1371/journal.pone.0201388

**Published:** 2018-07-26

**Authors:** Veronique Deschodt-Arsac, Romain Lalanne, Beatrice Spiluttini, Claire Bertin, Laurent M. Arsac

**Affiliations:** 1 Université de Bordeaux, CNRS, Laboratoire IMS, UMR 5218, Talence, France; 2 Université de Bordeaux, Faculté des STAPS, Pessac, France; 3 URGOTECH, 15 avenue d’Iéna, Paris, France; Pondicherry Institute of Medical Sciences, INDIA

## Abstract

**Introduction:**

Heart rate variability biofeedback (HRV-BFB) training, a method whereby one controls an unusually low breathing rate to reach cardiac coherence, has been shown to reduce anxiety and improve cardiac autonomic markers in diseased people, but much less is known about HRV-BFB benefits in healthy people. Here we investigated potential benefits in young competitors experiencing stress during university examinations as well as persistence of benefits after HRV-BFB training cessation.

**Methods:**

A group of sports students (n = 12) practiced 5-min HRV-BFB training twice a day for 5-weeks using URGOfeel^®^ (URGOTECH) and was compared to a control group (n = 6). University examinations occurred immediately after HRV-BFB training (Exam1), then 12-weeks later (Exam2). Anxiety markers and cardiac autonomic markers were assessed at baseline, Exam1 and Exam2. Principal Component Analyses (PCA) that combined all these markers were computed at Exam1 and Exam2 to emphasize covariations.

**Results:**

At Exam 1, immediately after HRV-BFB training cessation, the experimental group demonstrated greater autonomic markers but similar states of anxiety when compared to the Control group. Twelve weeks later at Exam2, autonomic markers were greater and anxiety scores were lesser among the experimental group. PCA highlighted covariations only within cardiac autonomic markers at Exam1. Rather, variations in cardiac markers were associated with anxiety markers at Exam2.

**Conclusion:**

Short sessions of HRV-BFB training for a brief period of 5 weeks bring substantial benefits to autonomic markers and anxiety levels in young competitors. Here beneficial effects persisted for 12 weeks. Dissociated profiles of anxiety and cardiac autonomic adaptations shed new light on the role of the amygdala in heart-brain interactions after cardiac coherence training.

## Introduction

In recent years, brain-heart interactions suggested by Claude Bernard more than 150 years ago, have been clarified by the strengthening of both conceptual and physiological backgrounds. Cerebral arousal and autonomic control over the cardiovascular system influence one another. Thus, modifying one will influence the other and this may persist over time.

A complex and functional structure that supports heart-brain interactions has been conceptually described over the years and is consensually referred to as the central autonomic network (CAN) [1,2]. The conceptual design of the CAN states that cortical and medullar units could be involved [1], a statement which has recently received strong support thanks to functional imaging of brain structures [3]. These studies show a good adequacy between active areas of the brain and previously described CAN components [3,4]. Perhaps the most striking observation in heart-brain interactions is the demonstrated benefit for the brain that results from modulating cardiovascular dynamics by simply controlling one’s breathing pattern for a few minutes in quiet conditions [5]. It is now widely accepted that such practice helps to maintain parasympathetic cardiac modulation even in stressful conditions, which has been shown to be beneficial, mainly in chronically stressed subjects [6]. The practice is mostly based on experimental works by Lehrer [5], who described the resonant frequency breathing technique. The technique consists in superposing the frequencies of the two oscillators that modulate the prime pacemaker of the heart, the sinus node. This is achieved by slowing the frequency respiration—naturally around 0.25–0.35 Hz—that drives parasympathetic cardiac modulations, toward the natural operating frequency of the sympathetic drive, around 0.1 Hz. When perfect superposition is achieved, resonance occurs, which greatly rises the magnitude of the resulting oscillations [7] characterizing a state of cardiac coherence. Cardiac coherence yields greater reflex efficiency and afferent brain stimulation and an optimized psychophysiological state [8].

In the last two decades, interventions based on cardiac coherence practice have been successfully used to treat a variety of pain and anxiety-related conditions [9]. As well, cardiac coherence helps to maintain parasympathetic cardiac modulation in stressful conditions [6].

Although classical meditation or yoga techniques allow practitioners to reach a near resonant state, it is demonstrated that biofeedback routines are needed to obtain optimal results [10]. Such practice is referred to as heart rate variability biofeedback (HRV-BFB) or, when practiced regularly, HRV-BFB training.

The need for biofeedback is due to inter-individual differences in baroreflex frequency, which can vary from 0.07 to 0.11 Hz [7,11] corresponding to 4.5 to 6.5 cycles per minute [10]. To help practitioners obtain persistent benefits, a number of companies have purposely developed biofeedback equipment.

Most of the studies using HRV-BFB training have been conducted with patients suffering from chronic physiological [12] and psychological [8] diseases. In more recent years, benefits have been reported in people with acute stress disorder [13,14], thus encouraging the use of HRV-BFB training by people exposed to stress for improving health, well-being or performance. Typically, athletes are predisposed to cope regularly with stress due to successive competitions. To date, studies on potential benefits of HRV-BFB training in competitors are only in their initial stages [15]. One possible obstacle for the development of the practice in competitors might be the recommended duration of HRV-BFB sessions, which can go up to 40 minutes [7]. Better adherence could be expected with reduced duration, but the effectiveness of short sessions of HRV-BFB training, has not been demonstrated to date. Also, the extent to which the benefits of HRV-BFB training might persist over time after training cessation has received little attention [12]. And yet, this is a critical aspect for athletes and coaches who pay great attention to the periodization of training contents over days, weeks and months.

The present study was designed to examine the effects of five-weeks of HRV-BFB training on the potential benefits of heart-brain interactions. The originality of the present work lies in the fact that: i) both short-term effects and persistent effects of HRV-BFB training are investigated; ii) HRV-BFB training is based on short sessions (5 min) twice a day; iii) healthy and well-trained subjects are investigated.

## Materials and methods

### Participants

18 students (5 females) aged 20.5 years ± 1.5, were recruited during lectures at the Faculty of Sport Sciences of the University of Bordeaux. Three out of five females were taking oral contraceptive pills, and as such, natural hormonal variations were suppressed. The other two females measured their basal body temperature each morning and recorded the day of menses throughout the experimental period. Physiological measurements were taken in the follicular phase as days 6 to 10 after the first day of bleeding. Each female filled out the Menstrual Distress Questionnaire (MDQ), which evaluated physical, emotional, and behavioral symptoms during menstrual cycles. All of them presented a small score. Overall, the recorded information suggests little, if any disturbances linked to menstrual cycles [16,17].

Participants were randomly allocated to the experimental training group (HRV-BFB, n = 12) or the Control group (n = 6) before further information was given about the study. The study was approved by the institutional Review Board of South-West France and Overseas French departments, France. Written informed consent was obtained from all the volunteers. All the experiments respected the principles set by the CNIL (Commission Nationale de l’Informatique et des Libertés), the CPP (Comité de Protection des Personnes), and the ARS (Agence Régionale de Santé).

None of the participants were receiving medical treatment before enrollment. All the participants were well-trained athletes accustomed to stressful situations (6–10 training sessions per week). All participants were non-smokers. They were required to abstain from food or drink for 2 hours before the training procedure and from caffeine on the days of university exams to control for the impact of these variables on autonomic cardiovascular responses.

### Training procedures

**Fig 1:**
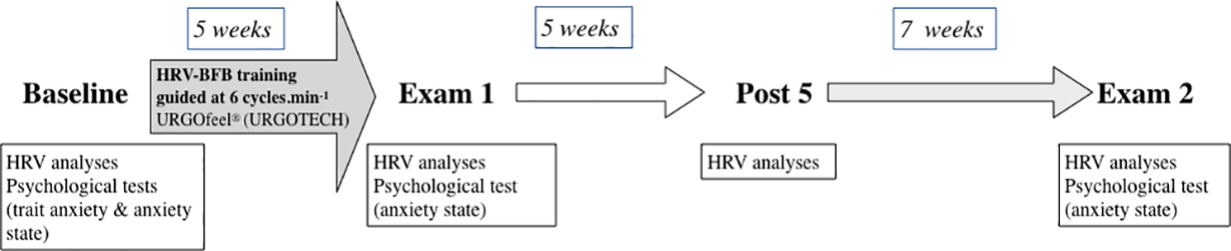
Schematic representation of the experimental procedure.

Heart rate variability biofeedback (HRV-BFB) training was assigned to the experimental group (HRV-BFB group) during 5 weeks (Fig 1). Subjects were asked to breathe at 6 cycles.min^-1^ for a 5-min period twice a day (morning and evening). For that, they were instructed to remain seated in a quiet environment at home during the same hours each day: 8:00–10:00 am and 7:00–10:00 pm.

**Fig 2:**
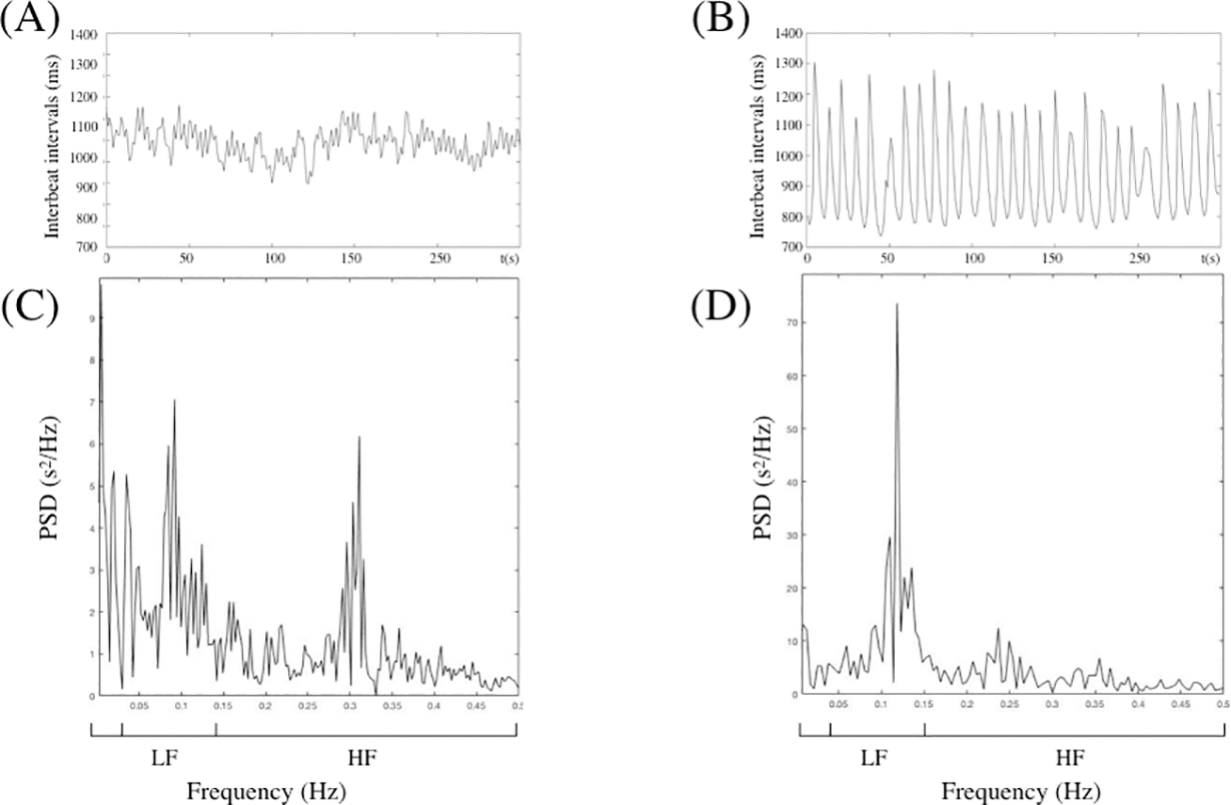
Individual data: Interbeat intervals and frequency analysis during rest periods with spontaneous breathing and during HRV-BFB training. (A) Example of interbeat intervals during four minutes of a resting state. Changes in RR are small and irregular. (B) Example of interbeat intervals during five minutes of HRV-BFB training. Changes in RR are large and regular due to respiration. (C) Frequency analysis during 5 minutes in a resting rate indicates low amplitude of L and HF. (D) Frequency analysis during 5 minutes of HRV-BFB training indicates very high amplitude LF oscillation at a single frequency.

Biofeedback operated thanks to a connected device developed by URGOTECH linked by Bluetooth at the smartphone application, URGOfeel^®^ (URGOTECH). This non-invasive device uses an infrared finger photoplethysmograph to measure inter-beat-intervals in the pulse rate. This was used to detect respiratory sinus arrhythmia (RSA) and to display HRV biofeedback in order to help control breathing rates at 6 cycles.min^-1^. At this frequency, respiratory effects on heart rate stimulate baroreflex effects, such that both respiratory sinus arrhythmia and baroreflex gain are maximized and consequently induce the highest amplitude of low-frequency HR oscillations. This frequency, named resonant frequency by Lehrer who first described this method [7], causes heart rate to go up and down in phase with respiration [11]. Fig 2 presents an example of HRV power spectrum obtained using Fast Fourier Transform (FFT) with a spontaneous breathing frequency (Fig 2A). In this case, the low frequency (LF) component occurs between 0.08 and 0.15 Hz and the high frequency (HF) component occurs between 0.30 and 0.40 Hz. When the subject breathed at 6 cycle.min^-1^ during HRV-BFB (Fig 2B), a very high oscillation amplitude was observed at the single frequency located near 0.1 Hz (Fig 2B).

During the intervention period, participants kept a daily diary about their training activities, diet, sleep quality, and personal feelings of fatigue. Since the subjects were trained athletes, we were particularly vigilant about these results especially about loss of sleep quality and/or overtraining syndrome that could appear during the HRV-BFB training phase and potentially may skew the results. No subject had more than 3 nights with under 6 hours of sleep or with very disturbed sleep. Subjects were asked to write down each score of cardiac coherence calculated from the URGOfeel^®^ application. This score was related to the capacity of the subject to follow indications from HRV biofeedback to optimize cardiac coherence.

### Psychological analysis

Participants filled out a series of questionnaires online to measure various aspects of stress and anxiety prior to HRV-BFB training (Baseline) and again after training, at the beginning of Exam1 then Exam2 (Fig 1). Anxiety and stress were measured with tests adapted from Skinner and Brewer [18] at baseline to detect individual anxiety trait and with the Spielberger’s State-Trait Anxiety Inventory (STAI) [19] to assess the level of anxiety state at the beginning of each exam session.

### Physiological analysis

Cardiac autonomic markers were assessed from recorded inter-beat-intervals (R-R intervals in milliseconds) for a minimum of 10min using a Polar H7 belt associated by Bluetooth with the application HRV Logger^®^. Subjects were tested four times for HRV: twice at rest (at baseline and after 5 weeks post-HRV-BFB training, Post5 see Fig 1) and twice during university exam sessions (Exam1 and Exam2, Fig 1). At rest, experiments were conducted in a quiet room with a constant temperature of 25 ± 2°C and a relative humidity of 60–70% and in a standardized seated position. Examination sessions were assumed to be a real-life stressor. Exam1 occurred at the end of the HRV-BFB training while Exam2 occurred 12 weeks after training cessation. During university examination, heart rate recordings began 10 min before the exam topics were handed out, and ended 10 min afterwards. To control for diurnal variations, HRV was always measured between 8 and 12 am.

### Time domain analysis

In time domain, we computed the mean interbeat interval (R-R) and the square root of the mean of the sum of the squared differences between adjacent intervals (RMSSD). RMSSD was analyzed because it represents short-term HRV variability and reflects to a large extent high-frequency (vagal) autonomic modulations.

#### Frequency domain analysis

For HRV analysis in the frequency domain, each R-R interval series was converted to equidistant resampled (4Hz) series by using cubic spline interpolation. The spectral HRV markers were calculated from Fast Fourier Transform (FFT). Average spectral power was estimated within the low frequency (LF) (0.04–0.15 Hz) and high frequency (HF) (0.15–0.4) bands, which represent the influence of sympathetic and parasympathetic activity. From these markers, other indicators have been derived to quantify so-called sympathovagal balance: LF/HF, LF or HF in normalized units (HFnu) calculated as HF/(LF + HF). The latter three contain strictly identical information and HFnu was used in the present study. All procedures were conducted in accordance with the recommendations of the Task Force of the European Society of Cardiology and the North American Society of Pacing and Electrophysiology [20].

### Statistical analysis

Values were expressed as means ± standard error of the means. The normality of the data was tested with D’Agostino-Pearson normality test. Comparisons between groups (HRV-BFB group *vs*. Control group) were analyzed using repeated measures ANOVA followed by paired or unpaired *t-*tests with Bonferroni corrections for multiple comparisons (Prism 6, Graphpad software). Results were considered significant at *P*<0.05. Principal component analysis (PCA) is a mathematical procedure that transforms a number of (possibly) correlated variables into a (smaller) number of uncorrelated variables called principal components. PCA with varimax rotation were? computed using SPSS version 2.0.

## Results

### Baseline

Baseline psychological and physiological markers were similar in HRV-BFB group and in Control group (Table 1). Trait anxiety in each group was defined by the same dynamics of threat and challenge appraisals. Therefore, subsequent effects of HRV-BFB training can be unambiguously detected in the HRV-BFB group.

**Table 1:** Baseline psychological (trait anxiety) and physiological, temporal and frequency indices obtained for the both experimental groups.

	HRV-BFB group (n = 12)	Control group (n = 6)	P-value
Trait anxiety (Challenge)	25 ± 1.3	27 ± 2.4	0.3002
Trait anxiety (Threat)	31 ± 3.1	29 ± 5.3	0.7104
RMSSD	56 ± 4.7	52 ± 4.3	0.5929
LF/HF	3.1 ± 0.3	4.2 ± 0.5	0.0524

Values are expressed as means ± standard error of means. Analysis done by unpaired student ‘t’ test with Bonferroni corrections. No significant differences (Results were considered significant at *P*<0.05) were found between the HRV-BFB group and the Control group.

### HRV-BFB training follow-up

**Fig 3:**
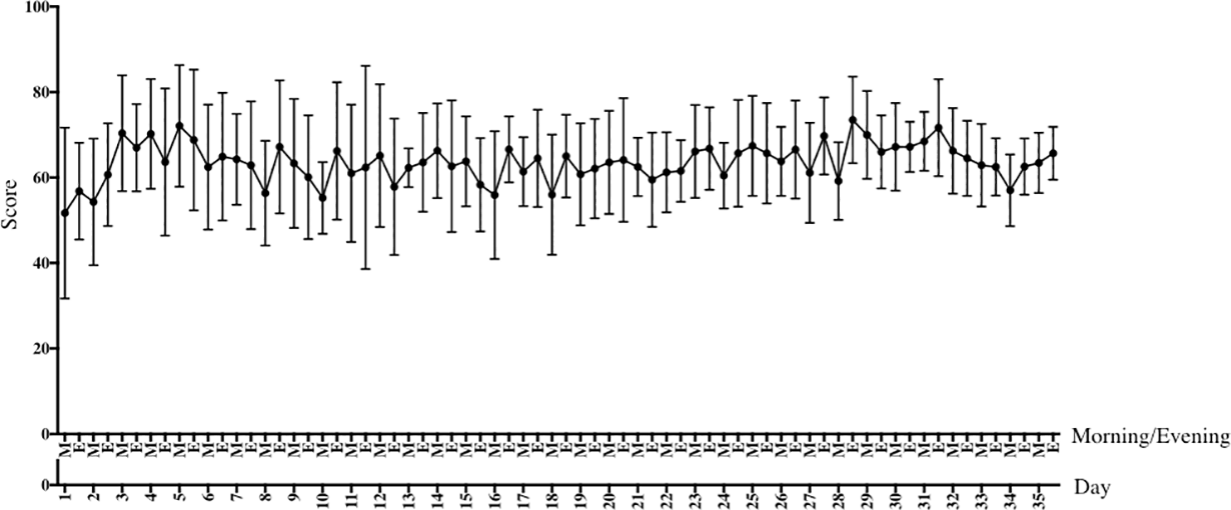
Mean (standard error) cardiac coherence scores obtained from each HRV-BFB training session (morning, M and evening, E) recorded with the application URGOfeel^®^ (URGOTECH).

Fig 3 shows the time course of averaged cardiac coherence scores along the training period. Individual scores illustrate how each subject reached the targeted breathing rate thanks to biofeedback during daily training sessions. Typically, these scores increased during the first days of training practice, thus pointing to the progressive habituation to breath at an unfamiliar slow rate.

#### Psychological training effects

**Fig 4:**
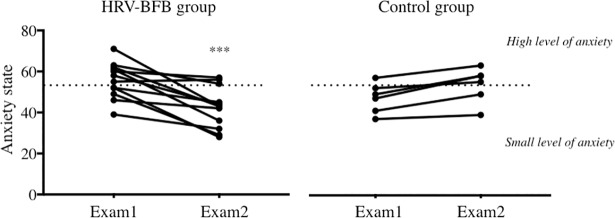
Individual anxiety states in each group at exam1 and exam2. Note the significant decrease in anxiety in the HRV-BFB group only. The dotted line represents high levels of anxiety state (data from Spielberger D. 1966). *** p < 0.001 between the two sessions of examination.

There was no difference in scores of anxiety state at Exam1 between groups (Fig 4) in spite of 5-weeks of HRV-BFB training in the experimental group. By contrast, a clear difference appeared at Exam2, 12-weeks after training cessation, where anxiety state dropped in the HRV-BFB trained subjects (-23.3% ± 16.1%, Fig 4). It is worth noting that all HRV-BFB subjects except one had a lower anxiety state and that 3 out of 12 students presented a drop >40%. This points to delayed benefits of HRV-BFB training in our conditions.

By contrast, anxiety states remained high both at Exam1 and at Exam2 in control subjects.

#### Physiological training effects

**Fig 5:**
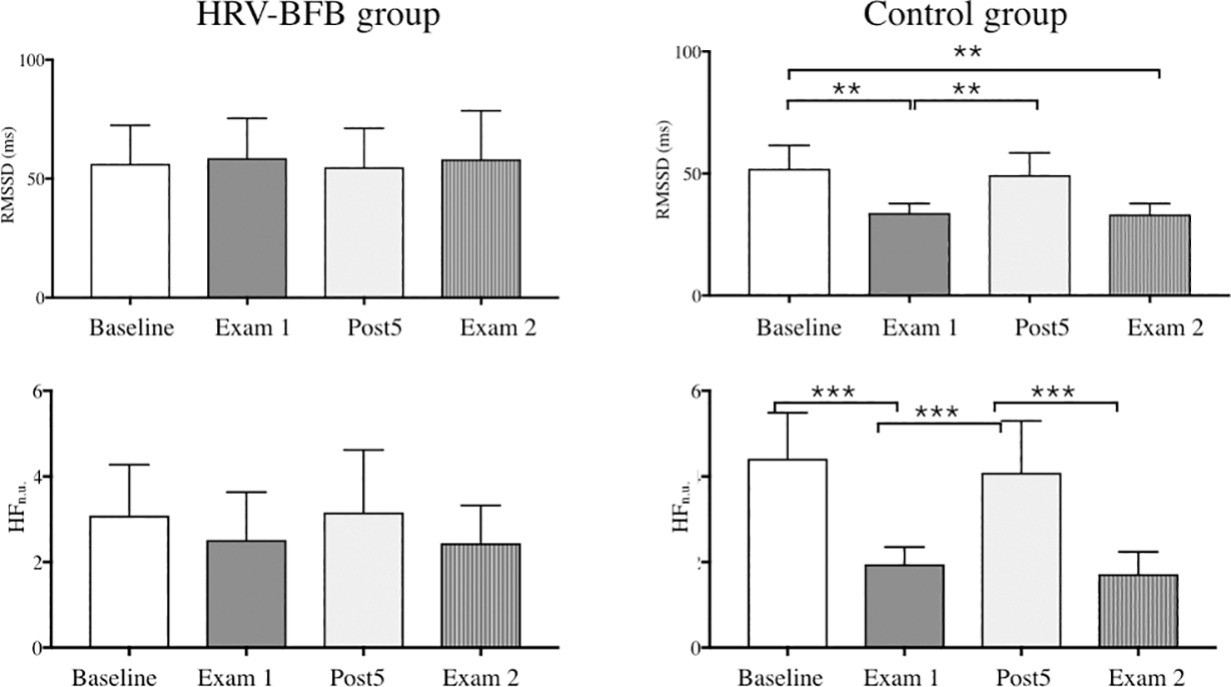
Autonomic function evaluated by HRV analysis in time and frequency domains at baseline, at 5 weeks post BFB-HRV training (Post5), and at Exam1 and Exam2. Mean values of RMSSD were reported at the top of the Figure. Sympathovagal balance calculated from HF in normalized units (HFnu) was reported at the bottom of the Figure. *** p < 0.001; ** p < 0.01 indicate significant differences between sessions.

It is worthy of note that cardiac autonomic markers, both in time (RMSSD) and frequency (HFnu) domains, were not affected by the stress of the examinations (Exam1 and Exam2) in the HRV-BFB group (Fig 5), since values were similar to those obtained at baseline (Fig 5). By contrast, in the Control group each exam session induced a drop in cardiac autonomic markers when compared to baseline (Fig 5).

An additional assessment of cardiac autonomic markers was scheduled 5-weeks after HRV-BFB training in resting conditions. In each group, similar RMMSD and HFnu were obtained at that time (Post5), which also corresponded to baseline values. This discards any drift in cardiac autonomic control at rest in the trained group and rather highlights adaptations in the specific response to stress.

### Dimension reduction: Principal Component Analysis (PCA)

**Fig 6:**
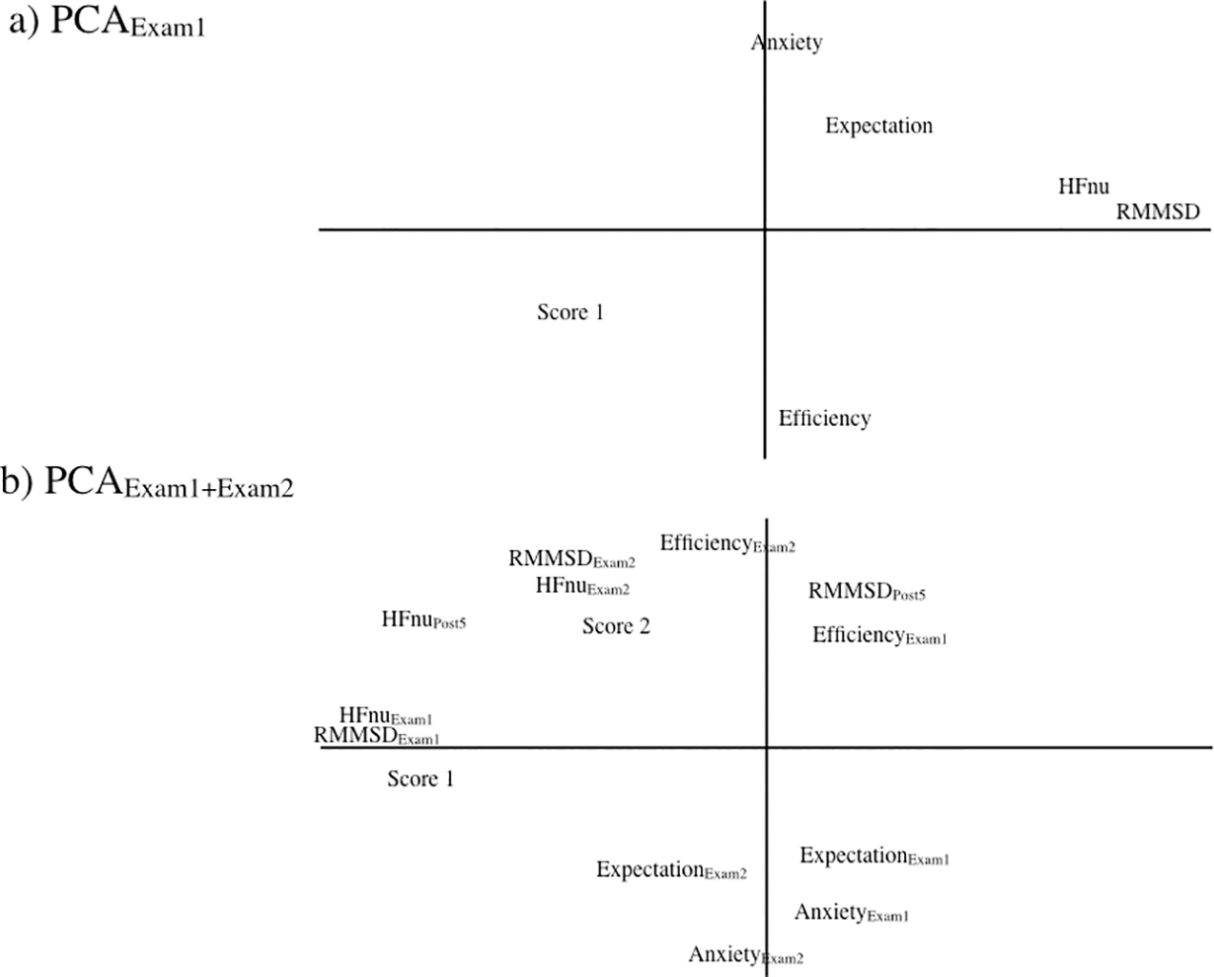
Principal component analysis for BFB-HRV group. (A) data recorded at Exam1, (B) data recorded at Exam1 and at Exam2.

PCA was used here to emphasize variations and bring out patterns in our dataset for the HRV-BFB group that combined psychological markers (Anxiety, Expectation, Efficiency), autonomic markers (RMSSD, HFnu) and exam performance (Score1, Score2). Two principal component analyses (PCA _Exam1_ and PCA_Exam1+Exam2_) with varimax rotation were computed (Fig 6).

As regards Exam1 (Fig 6A), the first factor in PCA explained 32% of the variance and encompassed RMSSD and HFnu. The second factor explained about 25% of the variance and encompassed Anxiety and Efficiency, a component of anxiety state.

As regards Exam2 (Fig 6B), the first factor that explained 35% of the variance encompassed autonomic markers RMMSD_Exam1_ and HFnu_Exam1_. The second factor explained about 23% of the variance and pointed to covariations in autonomic and psychological markers as it was defined by Anxiety_Exam2_, Efficiency_Exam2_, RMSSD_Exam2_ and HFnu_Exam2_. So, in contrast with Exam1, psychological and physiological markers at Exam2 showed covariations.

## Discussion

The present study yields four original pieces of information about using heart rate variability biofeedback training. First, even short lasting daily HRV-BFB training sessions (5-min twice a day) demonstrated benefits on cardiac autonomic control and anxiety during stress. Second, young and healthy subjects, habituated to coping with stress associated with a sports competition demonstrated these positive effects. Three, HRV-BFB training effects persisted several (12) weeks after practice cessation. Finally, psychological and cardiac autonomic adaptations to HRV-BFB training showed different profiles over the weeks, which could shed light on heart-brain interaction dynamics.

Lehrer developed the resonant frequency breathing technique about two decades ago, and since then there has been substantial support for heart rate variability biofeedback as an efficient treatment for a number of disorders [21,22]. In addition, while less well documented, there is evidence for better athletic performance after training in HRV-BFB, pointing to potential positive effects in healthy people as well [15,23,24].

In more recent years, links have become increasingly obvious between baroreflex resonance, functioning of so-called central autonomic network or CAN [1,25] and positive effects of HRV-BFB on the brain. The CAN describes an interconnected network that includes the prefrontal, cingulate and insula cortices exchanging a bi-directional flux of information with the amygdala. The agmydala, a center for emotional control, communicates directly with the nucleus tractus solitaris located in the brain stem, through which the baroreflex is mediated. It is believed that the existence of such interconnections could explain why HRV-BFB training of baroreflex resonance could have positive effects for treating anxiety. Our results provide additional support for such top-down and bottom-up functioning, given that HRV-BFB training improved anxiety states during exams (Exam2 only, which is a critical observation discussed below) and that such changes were associated with enhanced cardiac autonomic control.

The design of the HRV-BFB training seems critical in obtaining obvious benefits. Lehrer et al. [7] showed improved vagal (parasympathetic) afferents in sedentary adults after ten sessions of 40min of biofeedback HRV training. In subjects with major depressive disorders, significant effects arose after four sessions, thus pointing to the need of regular and sustained training [26]. Recently, Gross [27] used a five-session 3–5 min protocol in sport support staff and did not obtain improved basal autonomic control. While a sufficient time dedicated to HRV-BFB training seems necessary for significant improvements, in the present study we showed that short 5-min sessions twice a day is powerful in young competitors. We suggest that athletes demonstrate specific responses, which could make even short-term protocols efficient enough. This remains to be further explored, keeping in mind the interest of short lasting protocols in elite athletes to promote better adherence.

Exam-related anxiety is experienced by many students [28,29]. The interconnected network described above supports the idea that this experience is potentially reflected in cardiac autonomic control. In the present study, the values of autonomic markers dropped during Exam1 in the control group while HRV-BFB trained subjects maintained values similar to baseline (Fig 5). Interestingly, this adaptation to HRV-BFB was associated with better exam scores (Fig 6B), thus demonstrating positive effects of cardiac coherence training on both autonomic control and mental performance when exposed to stress.

A striking observation immediately after training was the dissociation between psychological and cardiac autonomic responses. This was illustrated by the absence of difference between groups in the anxiety state at Exam1 despite different HRV profiles at this stage. The orthogonality between psychological and physiological markers highlighted by PCA_Exam1_ strengthens the existence of independent regulations. Within the framework of the neurovisceral integration model (CAN) that supports interconnectivity, one should expect concomitant rather than dissociated psychological and cardiac autonomic responses. This apparent discrepancy could be explained by the very mechanisms supporting connections in the CAN and the critical role played by the amygdala. Recent neuroimaging studies shed additional light on neurocognitive mechanisms of anxiety [3,30,31]. It is suggested that the balance of activity within the amygdala-prefrontal circuitry is altered by anxiety [32]. One could suggest that high level of anxiety, as quantified here just at Exam1 disrupts this circuitry, so that psychological marks reached saturation. This is reflected in pretty high anxiety state scores at Exam1 amounting to 40–65 which is as high as scores classically obtained in control subjects in similar conditions [29].

Going one step further, our protocol was designed to assess the effect of HRV-BFB training 12-weeks after practice cessation, at Exam2. Physiological benefits of HRV-BFB were still present at Exam2 as reflected in maintained cardiac vagal markers in response to the exams stressor. It is unlikely that this persistence in autonomic adaptations could be a bias, considering that people in the control group demonstrated a drop in autonomic markers similar to the one observed during Exam1. It could be added that this drop is a specific response to the stress induced by academic examination since intermediate assessments of autonomic markers at rest, 5-weeks after training cessation, indicated normal autonomic control.

To the best of our knowledge, the maintenance of HRV benefits many weeks after HRV-BFB training cessation is an original observation, which is not reflected in the intermediate measures at rest 5-weeks after cessation, and could point therefore to one’s capacity to retain resistance to stressful situations, even in the absence of visible improvements of autonomic control at rest. This observation is not trivial in the context of the present study that enrolled young competitors, habituated to coping repeatedly with stress during major sport events, and who obviously improved their stress management durably when using an abbreviated training session of cardiac coherence.

Distinct time courses in physiological and psychological benefits to HRV-BFB training—early cardiac autonomic improvements but delayed reduction of anxiety–may shed light on the functioning responsible for heart-brain interactions in this context. While PCA_Exam1_ indicated orthogonality between cardiac autonomic markers and anxiety, by contrast, PCA_Exam2_ demonstrated covariations between HRV and anxiety markers. At this stage, the observed drop in anxiety in HRV-BFB trained people could favor the withdrawal of the influence of the amygdala on the CAN circuitry. When this withdrawal operates, heart-brain interconnectivity is supposed to be restored, which could explain concomitant rather than dissociated psychological and cardiac autonomic responses during examination.

The present study is not without limitations. Firstly, self-report questionnaires are reliable but might provide imperfect measures of anxiety. All psychological measures have measurement errors due to a variety of known causes as well as random effects [33].

Secondly, because of complex fluctuations in HRV, cardiac autonomic markers may be difficult to assess with classic linear methods [29,34]. Nevertheless, most of the studies on heart-brain interaction in Humans have extensively focused on vagal (parasympathetic) markers in time and/or frequency domains [35–37]. Typically, non-linear methods and complexity markers (*e*.*g*. entropy, fractal (scaling) exponents) bring no additional clues on vagal modulations of the heart and their computation requires long lasting HR measurements. In a large extent, fractal computations in the short-range domain are equivalent to frequency analyses [38].

## Conclusion

In conclusion, this study shows that anxiety states and autonomic function may be improved by short-lasting cardiac coherence training, which brings improved resistance to stressful situations, as shown here in young competitors. The absence of concomitant improvements in psychological and physiological adaptations in early weeks followed by concomitant improvements some weeks later, where HRV-BFB benefits persisted may shed new light on the role of the amygdala in heart-brain interactions.
